# Multi-Objective Optimization of Material Removal Characteristics for Robot Polishing of Ti-6Al-4V

**DOI:** 10.3390/mi17020146

**Published:** 2026-01-23

**Authors:** Fengjun Chen, Rui Bao, Meiling Du, Mu Cheng, Jiehong Peng

**Affiliations:** 1 School of Robot Engineering, Wenzhou University of Technology, Wenzhou 325000, China; 2 College of Mechanical and Vehicle Engineering, Hunan University, Changsha 410082, China; baorui@hnu.edu.cn (R.B.); jiehongjjj@hotmail.com (J.P.); 3 School of Mechanical and Electrical Engineering, Wenzhou University, Wenzhou 325035, China; dumeiling@stu.wzu.edu.cn; 4 Wenzhou Yihua Connector Co., Ltd., Yihua New Park, Huaxing Road, Wengyang Street, Yueqing 325606, China; chengmu@czt.com.cn

**Keywords:** robot polishing, material removal, roughness prediction, multi-objective particle swarm optimization (MOPSO), parameter optimization

## Abstract

This study employs a multi-objective particle swarm optimization (MOPSO) algorithm to address the dual-objective challenge in the robotic polishing of Ti-6Al-4V. The aim is to determine optimal parameters that minimize surface roughness while maximizing the material removal rate (MRR), thereby improving both surface quality and processing efficiency. First, a material removal depth model for end-face polishing is established based on Preston’s equation and theoretical analysis, from which the MRR model is derived. Subsequently, orthogonal experiments are conducted to investigate the influence of process parameters and their interactions on surface roughness, followed by the development of a quadratic polynomial roughness prediction model. Analysis of variance (ANOVA) and model validation confirm the model’s reliability. Finally, the MOPSO algorithm is applied to obtain the Pareto optimal solution set, yielding the optimal parameter combination. Experimental results demonstrate that at a normal contact force of 7.58 N, a feed rate of 4.52 mm/s, and a spindle speed of 5851 rpm, the achieved MRR and Ra values are 0.2197 mm^3^/s and 0.291 μm, respectively. These results exhibit errors of only 5.64% and 2.65% compared to model predictions, validating the proposed method’s effectiveness.

## 1. Introduction

Robot polishing offers significant advantages over manual methods by enabling the precise control of critical parameters (pressure, feed rate, spindle speed), which enhances efficiency while ensuring superior contour accuracy and surface quality. During robot polishing, the two key performance indicators of the material removal rate and surface roughness are significantly influenced by multiple process parameters. Consequently, establishing an accurate mathematical model for quantitative analysis and optimization is essential to maximize robotic polishing performance.

Research on polishing parameter optimization typically focuses on two primary and often conflicting objectives: maximizing the material removal rate to enhance polishing efficiency, and minimizing surface roughness to improve machining quality. Establishing precise predictive models for these objectives is critical. Existing material removal rate (MRR) models have been developed primarily for radial grinding tools, where tool–workpiece contact can be approximated using Hertzian contact theory. Chu et al. [1] developed a macroscopic material removal model by analyzing abrasive grain geometry microscopically and integrating Hertzian contact theory to determine contact pressure. Tian et al. [2] derived the contact model between the grinding wheel and the blade tip and the material removal model based on the elastic contact and Preston theory. For end-face grinding, Xiao et al. [3] derived a power-law contact force equation via geometric disk–workpiece analysis, validated through simulations. Xu et al. [4] addressed large-deformation flexible workpieces by coupling a nonlinear flexible contact model with Preston’s equations. In contrast, surface roughness lacks explicit physical models, with current approaches relying on empirical fits or data-driven methods. While power-law regressions [5] offer simplicity, second-order polynomials [6,7,8] improve accuracy. Neural networks [9,10,11] enhance predictions but face overfitting risks due to limited datasets.

Single-objective optimization typically employs direct modeling or Taguchi methods. Li et al. [12] optimized grinding pressure distribution using localized removal criteria, reducing contour errors without compromising roughness. Zhang et al. [13] quantified grinding depth point-by-point via force calculations at fixed feed rates. For roughness optimization, Li et al. [6] combined Taguchi and regression analysis, identifying rotational speed as the dominant parameter. Mohsin et al. [14] applied Taguchi–analysis of variance (ANOVA) to evaluate parameter effects across geometric workpieces, whereas Bai et al. [15] used particle swarm optimization to minimize roughness while preserving model accuracy. Sun et al. [16] employed the Taguchi method to determine the influence of four process parameters, including grinding wheel speed, feed rate, ingot rotation speed, and feed displacement, on the surface roughness of silicon carbide ingots, and identified the optimal set of process parameters.

Optimizing polishing processes is a multi-objective problem. Reducing it to a single objective cannot simultaneously maximize both efficiency and quality, posing challenges difficult for traditional experiments. Advances in artificial intelligence have led to the maturation of multi-objective optimization algorithms. Wang et al. [17], based on gray relational analysis, obtained the optimal parameters for the magnetorheological finishing of polymethyl methacrylate (PMMA) workpieces. After polishing, the surface roughness saw a reduction rate of 97.06%, while the material removal rate increased by 3.5% compared to that before optimization. Guo et al. [18] integrated gray relational analysis, Taguchi, and response surface methodology for Ti-6Al-4V blades, reducing roughness by 6.29% and increasing MRR by 16.11%. Xiang et al. [19] coupled broad learning system predictions with an improved grey wolf optimizer for automated parameter selection. Li et al. [20] hybridized long short-term memory—multilayer perceptron (LSTM-MLP) networks with non-dominated sorting genetic algorithm II (NSGA-II) to optimize robotic grinding parameters for roughness and time. Yang et al. [21] enhanced NSGA-II to concurrently minimize carbon emissions, processing time, and roughness. For cylindrical grinding, Alavijeh et al. [22] demonstrated that MOPSO and NSGA-II achieved similar Pareto fronts. Huang et al. [23] applied multi-objective particle swarm optimization (MOPSO) to optimize ultrasonic vibration-assisted grinding parameters after correlating inputs with forces and surface finish. In the current practice of industrial robotic polishing of Ti-6Al-4V using compliant tools, process parameter selection remains largely experience-based. Furthermore, existing optimization studies predominantly focus on belt grinding or radial grinding scenarios, or rely on purely empirical surrogate models, failing to provide a physics-informed, decision-oriented parameter selection methodology for axial grinding applications involving tilted rubber polishing disks.

Addressing this gap, this study focuses on robotic constant force end-face polishing of Ti-6Al-4V, constructing a physics-based and data-driven multi-objective parameter selection framework. The main contributions of this work are as follows: (1) Establishing a mechanics-based material removal profile and rate model for tilted flexible-disk polishing under constant normal contact force. The nonlinear stiffness of the rubber material is incorporated into the physical model and validated via finite element simulation. After calibrating model parameters, a material removal model based on controllable process variables applicable to real robotic polishing conditions is established. (2) Developing a roughness prediction model that reveals the main effects and interactions of normal contact force, feed rate, and spindle speed on Ra, providing an interpretable tool for roughness prediction and sensitivity analysis. (3) Integrating the above mechanistic MRR model and roughness regression model into the MOPSO algorithm. The resulting Pareto front is categorized into efficiency-dominant, balanced, and quality-dominant regions, facilitating rapid decision-making by engineers for different production tasks and reducing trial-and-error costs.

## 2. Material Removal Model

### 2.1. Removal Depth Model for End-Face Polishing Tool

**Figure 1:**
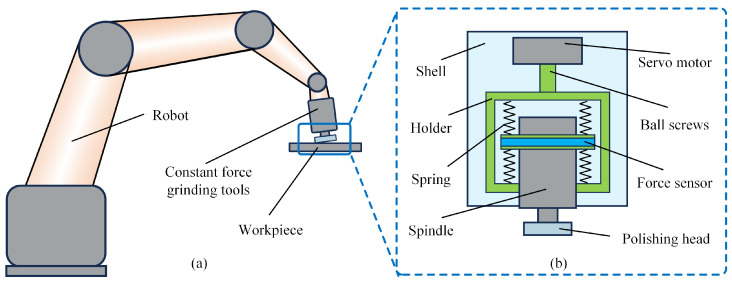
(**a**) Schematic diagram of robot constant force end-face polishing and (**b**) the constant force end effector.

Robot polishing employs two primary processing methods: radial polishing and end-face polishing. Figure 1 illustrates the robotic constant force polishing apparatus. In end-face polishing, the rotational axis of the polishing tool remains nearly perpendicular to the workpiece surface, enabling efficient processing of larger surface areas. During operation, the abrasive tool maintains constant-pressure contact with the workpiece surface, generating a cutting effect through high-speed rotation. Based on Preston’s equation, the instantaneous material removal depth at any contact point can be expressed as follows:

(1) $$dh=k_{p}pvdt$$

In this equation, $k_{p}$ represents the Preston coefficient, a parameter dependent on material hardness, processing conditions, and abrasive characteristics; p (Pa) denotes the local contact pressure; and v (mm/s) corresponds to the relative velocity between the tool and workpiece at the given point. In the apparatus used in this paper, the normal contact force is the primary, actively controlled component, and the normal pressure determines the abrasive grain penetration depth. The tangential force, being a passive effect resulting from the contact interaction and subject to variation with changing contact characteristics, contributes less significantly to material removal. Therefore, the contact pressure referred to in this context denotes the normal contact pressure.

**Figure 2:**
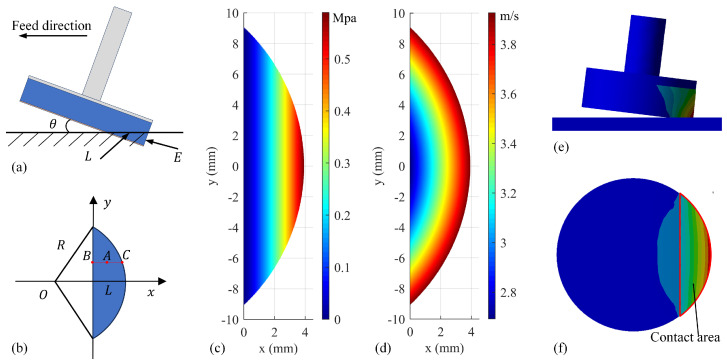
(**a**) Schematic of the contact between the polishing tool and the workpiece. (**b**) Simplified equivalent contact surface. (**c**) Pressure and (**d**) velocity distributions based on the analytical model. (**e**) Rubber deformation and (**f**) pressure distribution obtained from finite element simulation.

Figure 2a illustrates the contact geometry between the rubber disk polishing tool and workpiece during end-face polishing, where θ denotes the tool inclination angle relative to the workpiece surface, E (mm) represents the tool compression displacement, and L  (mm) indicates the maximum contact length along the *x*-axis direction, given by

(2) E=sinθ·L

Due to the tilt angle and rubber deformation, the contact contour between the tool and the workpiece on the right side of the *y*-axis is actually part of an ellipse. However, since the tilt angle is small, it is often approximated as a circle. Meanwhile, the left side of the *y*-axis exhibits minimal contact surface deformation and can generally be neglected. As a result, the final equivalent contact area is as shown in Figure 2b.

Considering the nonlinear elastic characteristics of rubber, where the equivalent stiffness exhibits a nonlinear relationship with compression displacement, the contact pressure at any point A(*x*,*y*) within the rubber disk–workpiece interface is modeled as a power function:

(3) $$p\left(x,y\right)=a{\left(e\right)}^{b}=a{\left(tan\theta \cdot x\right)}^{b}$$

where *e* (mm) is the compression of the rubber disk at a point, and *a* and *b* are constant coefficients of the power function, determinable through experimental calibration.

Since Equation (3) does not include the y term, the pressure distribution within the contact area primarily depends on the x-coordinate. Figure 2c illustrates the theoretical pressure distribution under ideal conditions. However, in actual polishing, slight pressure variations along the y-coordinate often occur due to non-uniform rubber deformation and geometric influences. To validate this conclusion, deformation and pressure distribution simulations were conducted in ANSYS 2022R1. The rubber disk was set with a 7° tilt angle and a 0.7 mm overall compression. The resulting deformation and pressure distribution are shown in Figure 2e,f. Within the contact region, the pressure contours obtained from finite element analysis exhibited slight concavity toward the left edge while remaining essentially parallel to the *y*-axis. The pressure demonstrated an approximately linear relationship with the x-coordinate, thereby confirming the accuracy of the conclusion.

**Figure 3:**
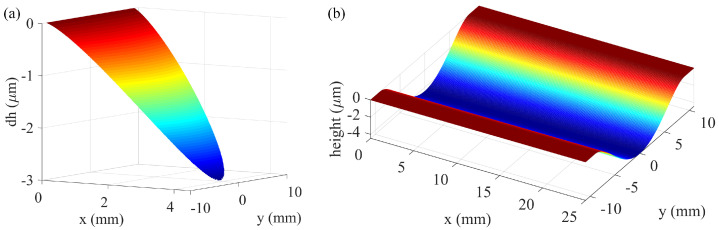
(**a**) Instantaneous and (**b**) cumulative material removal profiles.

The normal contact force is obtained by integrating the pressure distribution over the entire contact area:

(4) $$F_{N}=\iint p\left(x,y\right)ds=\int_{-y\_max}^{y\_max}\int_{0}^{f\left(y\right)}a{\left(tan\theta \cdot x\right)}^{b}dxdy$$

(5) $$y_{max}=\sqrt{R^{2}-{\left(R-L\right)}^{2}}$$

(6) $$f\left(y\right)=\sqrt{R^{2}-y^{2}}-R+L$$

where $y_{max}$ represents the maximum y-coordinate of the contact region, $f\left(y\right)$ describes the right-side contour profile of the contact area, and *R* is the radius of the polishing disk. In robot polishing, the contribution of feed velocity to the relative speed between tool and workpiece is typically negligible, as it is orders of magnitude smaller than the dominant spindle rotational speed.

(7) $$v\left(x,y\right)\approx v_{s}\left(x,y\right)=\frac{\pi n}{30}\sqrt{y^{2}+{\left(R-L+x\right)}^{2}}$$

where *n* is the spindle speed. Figure 2d illustrates the spatial distribution of the relative velocity magnitude across the contact zone. Substituting Equations (3) and (7) into Equation (1), the instantaneous depth of material removal at each point in the contact region can be derived, as shown in Figure 3a:

(8) $$dh=k_{p}a{\left(tan\theta \cdot x\right)}^{b}\cdot \frac{\pi n}{30}\sqrt{y^{2}+{\left(R-L+x\right)}^{2}}dt$$

Since $dt=dx/v_{f}$ and $v_{f}$ is the robot feed speed, the cumulative material removal depth at point A is given by

(9) $$h\left(y\right)=\int_{0}^{f\left(y\right)}dh=\frac{{\pi k}_{p}na}{30v_{f}}\int_{0}^{f\left(y\right)}{\left(tan\theta \cdot x\right)}^{b}\sqrt{y^{2}+{\left(R-L+x\right)}^{2}}dx$$

The integration of Equation (9) yields a solution independent of the *x*-coordinate, demonstrating that the cumulative material removal depth depends solely on the *y* position. The maximum material removal depth occurs at the centerline of the contact path, corresponding to y = 0. Substituting this condition into Equation (9) yields the following simplified formula:

(10) $$h_{max}=\frac{{\pi k}_{p}na({tan\theta )}^{b}}{30v_{f}}\cdot \frac{(bR+2R-L)L^{b+1}}{\left(b+1)(b+2\right)}$$

This formula explicitly establishes the direct relationship between key process parameters and the maximum removal depth, thereby enhancing the model’s practicality for real-world application and parameter adjustment. The maximum removal depth occurs at *y* = 0, corresponding to the centerline of the contact path. Figure 3b shows the final material removal curve, which can be seen to be similar to a parabolic shape, confirming this symmetrical distribution.

### 2.2. Material Removal Rate Model

The material removal rate (MRR) is defined as the volume of material removed per unit of time, calculated by multiplying the cross-sectional area of material removal by the feed velocity:

(11) $$MRR=S\cdot v_{f}=\frac{{\pi k}_{p}na}{30}\int_{-y\_max}^{y\_max}\int_{0}^{f\left(y\right)}{\left(tan\theta \cdot x\right)}^{b}\sqrt{y^{2}+{\left(R-L+x\right)}^{2}}dxdy$$

The equation contains three undetermined coefficients ($k_{p}$, a, and b), which are subsequently identified through experimental data. From the perspective of the grinding mechanism, the established material removal model aligns with the fundamental principles of abrasive processing. The increase in MRR with spindle speed and normal contact force can be explained mechanistically. A higher spindle speed increases the frequency of effective cutting actions per unit time, promoting more efficient micro-cutting. Conversely, a higher normal contact force increases the penetration depth of abrasive grains and the real contact area, shifting the material removal mode from primarily sliding and plowing to more efficient cutting, thereby increasing the volume of material removed by individual grains. The negligible influence of the feed rate on MRR stems from its minor contribution to the relative speed compared to spindle rotation. Its primary role is to distribute the total material removal over a longer path, without significantly altering the instantaneous cutting mechanism within the contact zone.

### 2.3. Material Removal Experiments

**Figure 4:**
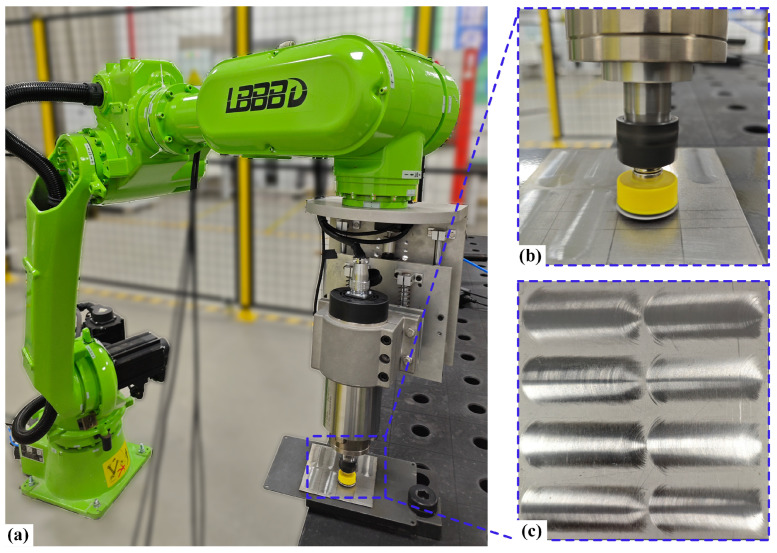
(**a**) Polishing robot device and (**b**) local polishing enlargement diagram (**c**) for polishing the Ti-6Al-4V workpieces.

The normal contact force during polishing critically determines both processing efficiency and surface quality. To achieve precise force control, we developed a robotic polishing system incorporating a specialized constant force end effector, as shown in Figure 4a. The system employs an LBBBD LT1850-D6 industrial robot (LBBBD Robot Co., Ltd., Dongguan, China) with 20 kg payload capacity and ±0.05 mm repeatability to execute tool path movements. A custom-designed force control module mounted at the robot’s end effector dynamically regulates the normal contact force within the 0–100 N range, while an integrated electric spindle provides rotational grinding action at speeds up to 24,000 rpm. As detailed in Figure 4b, the polishing setup features a 12.5 mm radius rubber disk with 10 mm thickness, mounted at 7° inclination to the workpiece surface. The selection of this inclination angle was based on preliminary experiments, aiming to achieve stable contact conditions, avoid stress concentration that could damage the polishing tool, and facilitate the evacuation of removed material. The process utilizes 600-grit abrasive paper on Ti-6Al-4V titanium alloy substrates, with the abrasive material specifically being aluminum oxide. Due to limitations in the experimental setup, a dry polishing method was employed here, with process parameters constrained to prevent the generation of excessive grinding heat. For applications targeting higher material removal rates, effective cooling strategies or tools with strong heat dissipation capabilities need to be integrated to mitigate thermal damage. Representative polished specimens are displayed in Figure 4c. Surface topography measurements were conducted using a SuperView W1-Lite optical 3D profilometer (Chotest Technology Inc., Shenzhen, China) to quantitatively evaluate polishing performance. This integrated system architecture combines robotic flexibility with precise force control capability for consistent polishing quality. To prevent the influence of abrasive tool wear on process stability and data consistency, a new sheet of abrasive paper was used for each independent polishing experiment in the subsequent tests.

**Figure 5:**
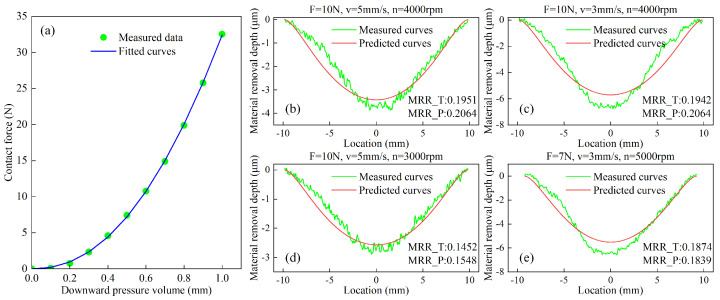
(**a**) Relationship between downward compression depth and normal contact force; (**b**–**e**) measured and fitted material removal depths.

To determine the equivalent stiffness coefficient of the rubber polishing disk, we measured the relationship between the vertical displacement E and normal contact force F. Experimental measurements of E and F revealed a nonlinear correlation, as shown in Figure 5a. Through least squares fitting of Equation (4) using MATLAB 2010b, the power-law coefficients were determined to be *a* = 0.4594 and *b* = 0.7305, characterizing the hyperelastic behavior of the rubber material.

To establish an accurate material removal depth model, we conducted multiple measurement experiments as shown in Figure 5b–e. The experimental data were processed using least squares fitting to identify the Preston coefficient $k_{p}$, yielding a mean value of $k_{p}$ = −0.004445. With all three unknown parameters in both the material removal profile model and removal rate model now determined, the complete MRR model can be expressed as

(12) $$MRR=0.000046216n\int_{-y\_max}^{y\_max}\int_{0}^{f\left(y\right)}x^{0.7305}\sqrt{y^{2}+{\left(12.5-L+x\right)}^{2}}dxdy$$

The figure compares the material removal rates obtained from experimental measurements (MRR_T) and model predictions (MRR_P), with units in mm^3^/s. The results show good agreement between the predicted and actual removal profiles. The mean absolute error for all data points of the removal profile is 0.3883 μm, and the average prediction error for MRR is 5.14%. As observed, the material removal rate increases with normal contact force and spindle speed, but is almost unaffected by changes in feed rate. However, some discrepancies are observed between the experimental and predicted removal profiles. As shown in Figure 5b, the measured removal profile is not perfectly symmetrical. This asymmetry may be caused by the slight angle of rotation of the spindle around the feed direction during the polishing process, resulting in uneven pressure distribution in the contact zone. Specifically, the pressure on the left side is slightly lower than on the right, resulting in a corresponding difference in material removal depth between the two sides. The measured material removal values along the central axis in Figure 5c,e show greater removal than predicted, primarily due to additional contact areas created by rubber deformation when the polishing disk is compressed. This phenomenon is also visible in the pressure distribution simulation of Figure 2f. Several other factors contribute to the observed profile errors: residual stains on workpiece surfaces introduce measurement errors; the model’s exclusive consideration of normal forces overlooks tangential and frictional effects; and high-speed spindle operations may induce tool–workpiece vibrations that perturb force control stability.

## 3. Roughness Prediction

### 3.1. Prediction Model

The surface roughness resulting from polishing is fundamentally determined by the microscopic interactions between abrasive grains and the workpiece material, exhibiting complex coupling effects with various process parameters. At the microscopic scale, lower roughness is achieved when material is removed via fine micro-cutting or plastic shearing within the plastic domain, leaving behind smaller pits or protrusions. Conversely, severe grain dislodgement and intense plowing effects, where material is pushed aside forming ridges, consequently increase roughness. Furthermore, unstable vibrations and excessive heat accumulation can also degrade surface quality. The relationship between surface roughness and various process parameters is inherently highly complex, making it difficult to derive an explicit mathematical expression directly from physical grinding models. Current approaches predominantly rely on empirical data to construct either regression models or neural network-based predictive models. Given the significant interaction effects and quadratic relationships among process parameters, simple linear functions prove inadequate for capturing the sophisticated nonlinear mappings between these parameters and surface roughness. To address this complexity, we employ a second-order polynomial regression model:

(13) $$Ra=\beta_{0}+\sum_{i=1}^{m}\beta_{i}x_{i}\;+\sum_{i=1}^{m}\beta_{ii}x_{i}^{2}+\sum_{i=1}^{m}\sum_{i<j}^{m}{\beta_{ij}x}_{i}x_{j}+\varepsilon$$

where *Ra* represents the predicted surface roughness. $x_{i}$ and $x_{j}$ represent the input process parameters, specifically normal contact force *F*, feed rate $v_{f}$ and spindle speed *n*. *m* is the number of input variables, where *m* is 3. $\beta_{0}$, $\beta_{i}$, $\beta_{ii}$, $\beta_{ij}$ are the coefficients of constant term, primary effect term, secondary effect term, and interaction term, respectively. ε accounts for the modeling error.

### 3.2. Parameters Calibration

To determine the coefficients of the surface roughness prediction model and investigate the influence of process parameters, a three-factor five-level orthogonal experiment was conducted. The first 25 parameter sets in Table 1 represent the orthogonal experimental design. The process parameter ranges for the orthogonal experiment were selected based on preliminary experiments and equipment specifications. A relatively low normal contact force was chosen to prevent excessive tool deformation and surface damage. The ranges for feed rate and spindle speed were determined to ensure stable operation of the setup, avoiding vibration issues during polishing while also preventing potential thermal problems at the upper operational limits. Prior to roughness measurements, all specimen surfaces underwent thorough cleaning to remove any contaminants that could affect measurement accuracy.

The model reliability was evaluated through analysis of variance (ANOVA), and the results are presented in Table 2. In the ANOVA framework, the F-value represents the ratio of between-group mean square to within-group mean square, quantifying the strength of the relationship between process parameters and target variables. Higher F values indicate greater parameter influence. The *p*-value serves as a statistical measure of parameter significance, where smaller values denote stronger statistical significance and better model fit. Following established conventions [24], terms with *p* ≤ 0.05 were considered statistically significant, while those with *p* ≤ 0.001 were deemed highly significant.

The ANOVA results demonstrate that the *Ra* regression model exhibits excellent statistical significance, with an F-value of 26.21 and *p* < 0.0001, indicating an extremely significant model with high fitting accuracy. The analysis reveals distinct significance levels among various terms: both A and AC show highly significant effects (*p* < 0.001), while B, A^2^, B^2^, and C^2^ demonstrate significant influence (*p* < 0.05). Notably, the AB interaction term (*p* = 0.3894) proves statistically insignificant and was eliminated from the model. Although the BC interaction shows marginal significance (0.05 < *p* < 0.10), suggesting some interactive effect, it may be conditionally retained. While the linear C term (spindle speed) alone appears insignificant (*p* > 0.1), its corresponding quadratic term’s significance necessitates its inclusion in the model. The observed statistical pattern, specifically an insignificant linear term coupled with a significant quadratic term for spindle speed, indicates a non-monotonic influence of spindle speed on Ra. Initially, within a certain range, increasing the spindle speed improves surface quality by enhancing the frequency of effective cutting actions. However, once the speed exceeds an optimal threshold, further increases induce significant vibrations and potentially excessive heat generation, consequently degrading surface quality. This non-monotonic relationship, characterized by initial improvement followed by deterioration, is often mathematically represented by an insignificant linear term alongside a highly significant quadratic term in the model. These findings collectively confirm strong nonlinear characteristics in the *Ra* regression model. After systematic refinement, the final surface roughness prediction model is expressed as

(14) $$Ra=0.4973925-0.0192763F-0.0176943v_{f}-0.0000680n+0.0000035Fn-0.0000021v_{f}n\;+0.000993F^{2}-0.0032061{v_{f}}^{2}+0.000000006n^{2}$$

**Figure 6:**
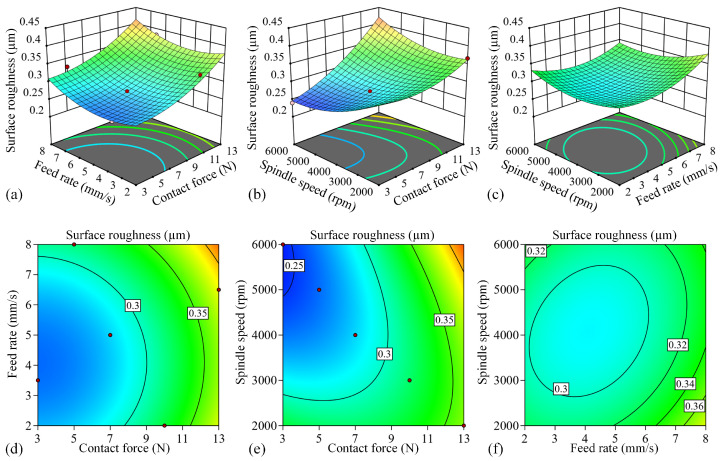
Response surface and contour plots of various process parameters and surface roughness. (**a**) Response surface of F and $v_{f}$; (**b**) response surface of F and n; (**c**) response surface of $v_{f}$ and n; (**d**) contour plot of F and $v_{f}$; (**e**) contour plot of F and n; (**f**) contour plot of $v_{f}$ and n.

Figure 6 presents the response surface plots and contour plots of the surface roughness model, illustrating the effects of process parameters on *Ra.* The response surface gradient reflects parameter sensitivity, with steeper slopes indicating stronger parameter influence. Similarly, the elliptical shape of the contour lines demonstrates more pronounced interaction effects between parameters.

Figure 6a illustrates that at a spindle speed of 4000 rpm, surface roughness (Ra) increases with both normal contact force and feed rate within the tested parameter range. This trend occurs because higher normal forces enhance the abrasive grain penetration depth, intensifying plowing effects and grain dislodgement while simultaneously promoting machining instability. Similarly, elevated feed speeds reduce the effective polishing time per abrasive grain, resulting in incomplete surface finishing and consequently higher roughness values. Figure 6d reveals nearly circular contours between normal contact force and feed rate, demonstrating their statistically insignificant interaction effect, consistent with the earlier ANOVA results. Figure 6b demonstrates that at a constant feed rate of 5 mm/s, surface roughness decreases with lower normal contact forces and higher spindle speeds. Elevated spindle speeds increase the frequency of abrasive–workpiece interactions per unit time, promoting more thorough material removal and consequently reducing roughness. However, when both contact force and spindle speed exceed optimal ranges, process-induced vibrations emerge, ultimately increasing surface roughness. The elliptical contours in Figure 6e exhibit pronounced elongation, demonstrating a significant interaction effect between normal contact force and spindle speed. This strong coupling suggests that variations in spindle speed substantially modify how normal contact force influences surface roughness. Figure 6c demonstrates that under a constant normal contact force of 8 N, surface roughness achieves its minimum value at intermediate feed rates and spindle speeds. This optimal processing window exists because extremely low feed rates coupled with high spindle speeds can lead to excessive heat accumulation despite providing more frequent abrasive contacts. The resultant temperature elevation negatively impacts grinding quality, causing surface roughness to increase. The elliptical contours in Figure 6f demonstrate a weak but measurable interaction between feed rate and spindle speed, as evidenced by their discernible curvature. Collectively, these response surface analyses reveal a pronounced quadratic relationship between surface roughness and the three process parameters, highlighting the complex nonlinear nature of the polishing process.

**Figure 7:**
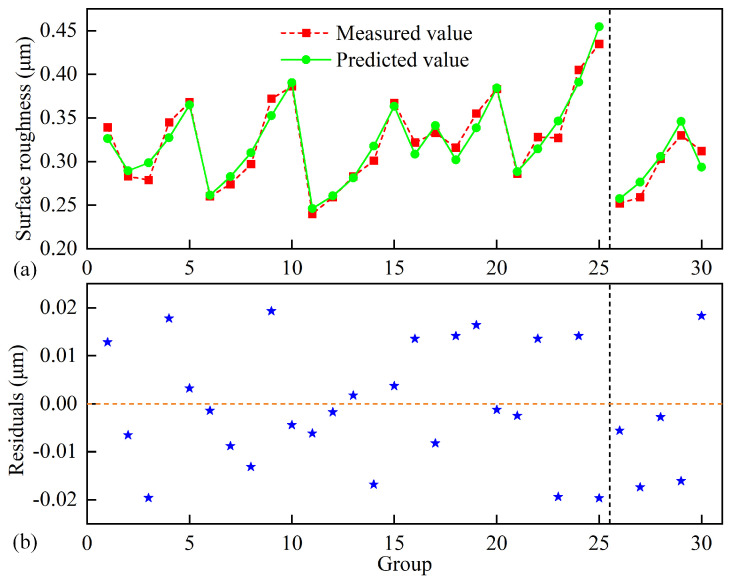
(**a**) Ra fitting and (**b**) residual plot of orthogonal experiments and verification groups.

The left side of the dashed line in Figure 7a demonstrates the refined model’s effectiveness in predicting surface roughness, with a calculated R^2^ value of 0.9371. This indicates that the regression model accounts for 93.71% of the variability observed in the orthogonal experiments, confirming its strong predictive capability. Furthermore, the residual plot shown on the left side of Figure 7b reveals that all residuals from the orthogonal experiments fall within a tight range of ±0.02 μm, with a uniform distribution pattern. The well-distributed residuals, combined with the model’s low root mean square error (RMSE) of 0.0154 μm, provide compelling evidence for the model’s accuracy and reliability in describing the experimental data.

To validate the accuracy of the surface roughness prediction model, five additional verification experiments were conducted, as shown in groups 25 to 30 in Table 1. As shown in the right sections of the dashed lines in Figure 7a,b, the model residuals remain consistently within ±0.02 μm for all validation tests. Quantitative analysis yields a residual sum of squares (RSS) of 0.00094 and a root mean square error (RMSE) of 0.0137 μm, with the model achieving an average prediction error of 4.12%. These results demonstrate that the roughness prediction model maintains reliable accuracy within the tested parameter ranges.

## 4. Process Parameters Optimization

### 4.1. Parameter Optimization Based on Multi-Objective Particle Swarm Optimization (MOPSO)

In polishing operations, MRR and Ra serve as key performance indicators for processing efficiency and quality, respectively. These two objectives typically exhibit a competing relationship, making process parameter optimization inherently multi-objective. The multi-objective particle swarm optimization (MOPSO) algorithm extends the conventional PSO approach to handle such conflicting objectives effectively. Firstly, the algorithm employs non-dominated sorting to identify solutions where neither MRR can be improved without worsening Ra, nor can Ra be improved without worsening MRR. These solutions form the Pareto front. Secondly, the algorithm maintains an external archive to store these best-performing non-dominated solutions throughout the search process. Finally, a crowding distance metric is used to preserve diversity within the archive. This ensures the Pareto front covers a broad spectrum of trade-offs, ranging from quality-optimal to efficiency-optimal performance. This systematic approach allows MOPSO to effectively explore the complex parameter space and provide a set of optimal compromise solutions.

Prior to implementing MOPSO for parameter optimization, the objective functions must be formally defined. These comprise the previously established mathematical models for the material removal rate and surface roughness. The optimization aims to identify an optimal set of process parameters that simultaneously maximizes MRR while minimizing Ra, with input variables comprising normal contact force, feed rate, and spindle speed. Since MOPSO operates as a minimization algorithm, the MRR objective function is transformed by taking its reciprocal.

(15) $$\left\{\begin{array}{c} \min\;f1=\frac{1}{MRR}\\ \min\;f2=Ra\; \end{array}\right.$$

Considering actual polishing conditions, the parameter ranges are constrained as follows: normal contact force *F*∈(3, 13) N, feed rate *v*∈(2, 8) mm/s, and spindle speed *n*∈(2000, 6000) rpm. These ranges serve as boundary conditions for the optimization problem.

**Figure 8:**
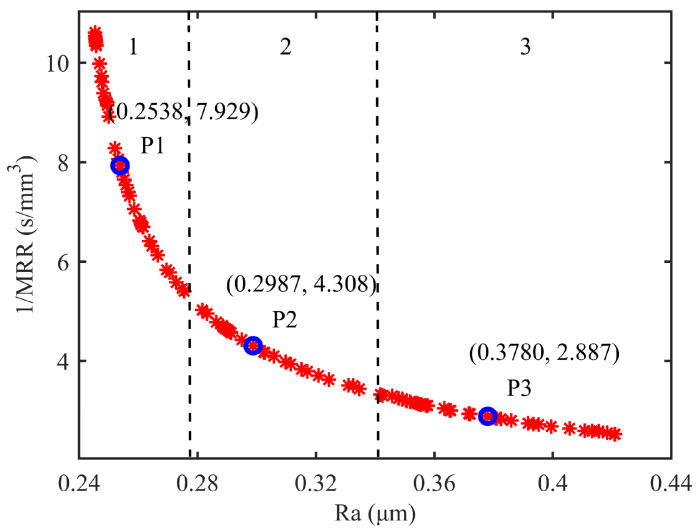
Pareto frontier solution set.

The MOPSO algorithm was implemented in MATLAB to optimize the process parameters. The key algorithm parameters were set as follows: the maximum number of iterations was 100, the population size was 100, the inertia weight damping ratio was 0.99, the inertia weight was 0.5, and the personal and global learning coefficients were 1 and 2, respectively. The resulting Pareto-optimal solutions are visualized in Figure 8, which displays the trade-off frontier between competing objectives in the solution space. This Pareto front represents the set of non-dominated solutions where neither objective can be improved without compromising the other. The leftmost endpoint of the Pareto front corresponds to the single-objective optimization result for minimizing roughness, while the rightmost endpoint corresponds to the single-objective optimization result for maximizing the material removal rate.

The Pareto front solution set reveals three characteristic regions with distinct trade-off behaviors between surface roughness and material removal rate. In the first region, the steep gradient indicates that minimal sacrifices in surface quality yield substantial gains in material removal efficiency, making this regime particularly advantageous for high-throughput polishing applications. Transitioning to the intermediate region, the more gradual slope demonstrates a balanced relationship where adjustments to either objective produce comparable effects, suitable for applications requiring compromise between processing speed and surface finish. The final region exhibits a shallow gradient where significant improvements in surface roughness can be achieved through relatively small concessions in the removal rate, proving most beneficial for precision finishing operations. Three points on the curve are uniformly selected as representative solutions for these three regions, and their corresponding results are shown in Figure 8. When a high surface quality is desired, point 1 can be selected as the optimal process parameter set. When a high machining efficiency is desired, point 3 can be selected. As the sandpaper is 600 mesh when polishing, which belongs to the semi-precision grinding stage, the surface quality and efficiency should be taken into account. So selecting the solution at the middle part of the curve is more in line with the actual overall objective, which is point 2. The optimal process parameter set is finally selected as follows: the normal contact force F is 7.58 N, the feed rate is 4.52 mm/s, and the main spindle rotates at 5851 rpm. For this combination, the predicted value of the material removal rate is 0.2321 mm^3^/s, and surface roughness is 0.2987 μm. This parameter combination ensures effective abrasive penetration and a high frequency of abrasive–workpiece interactions, while avoiding process instability or thermal effects caused by excessive conditions, thereby achieving a balance between MRR and Ra.

### 4.2. Experimental Verification

**Figure 9:**
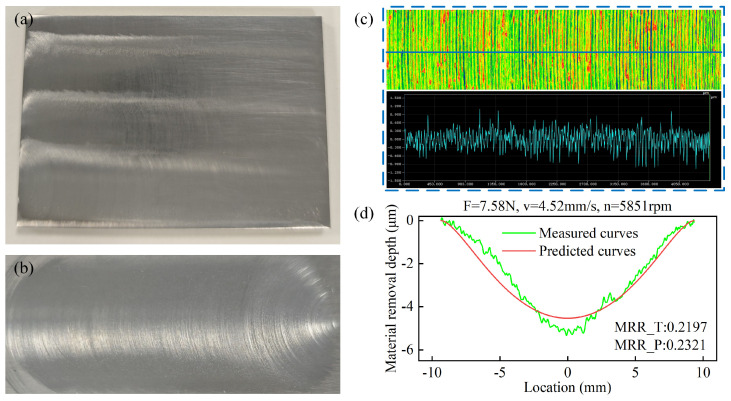
(**a**) Workpiece polished using optimal process parameters and (**b**) samples polished using a single polishing path; (**c**) roughness measurements and (**d**) material removal data.

To verify the accuracy of the proposed method, polishing experiments were performed using the optimal process parameter set obtained via the MOPSO algorithm. The resulting workpiece is shown in Figure 9a, while the surface quality achieved with a single polishing path is presented in Figure 9b. The simulation predictions were then compared against the actual measurements. The experimental results show excellent agreement with model predictions, with measured values of 0.2197 mm^3^/s for MRR and 0.291 μm for Ra. The experimental measurements indicate that the mean absolute error of the material removal profile is 0.3575 μm. The MRR shows only a 5.64% deviation from the predicted value, while the Ra deviation is 2.65%. This further confirms the high accuracy of the predictive models. The close correspondence between predicted and actual performance metrics substantiates the effectiveness of the proposed parameter optimization approach for robotic polishing applications.

## 5. Conclusions

To address the challenge of optimizing both surface quality and material removal efficiency in robotic polishing of Ti-6Al-4V titanium alloy, this study developed material removal and surface roughness prediction models. The research focused on multi-objective optimization of key process parameters including normal contact force, feed rate, and spindle speed to achieve optimal polishing performance.

(1) A material removal profile and rate models for disk polishing tools was developed. Finite element analysis revealed that the pressure distribution along the x-coordinate direction in the contact area was linear, and the final material removal contour closely resembles a parabola. Experiments showed a mean absolute prediction error of 0.3883 μm for the material removal profile. This provides a crucial model foundation for subsequent polishing path planning aimed at achieving high-precision dimensional control. The MRR model demonstrated 5.14% average prediction error in experimental validation.
(2) A surface roughness prediction model was established. Orthogonal experiments revealed significant parameter interactions. There was almost no interaction between F and v, while v and n exhibited a certain degree of interaction, and F and n showed significant coupling. The model achieved an R^2^ of 0.9371 with residuals within ±0.02 μm. Verification tests confirmed model robustness, showing 0.0137 μm RMSE and 4.12% average error.
(3) MOPSO-based parameter optimization was implemented. The optimal parameter set (*F* = 7.58 N, *v* = 4.52 mm/s, *n* = 5851 rpm) yielded a MRR of 0.2197 mm^3^/s and *Ra* of 0.291 μm, with prediction errors of 5.64% and 2.65%, respectively. This parameter combination effectively achieves simultaneous improvement in both material removal efficiency and surface finish quality, demonstrating significant practical value for industrial polishing applications.

## Figures and Tables

**Table 1:** Polishing experiments.

Group	F (N)	$v_{f}$ (mm/s)	n (rpm)	*Ra* (μm)	Group	F (N)	$v_{f}$ (mm/s)	n (rpm)	*Ra* (μm)
1	3	2	2000	0.339	16	3	6.5	3000	0.322
2	5	2	6000	0.283	17	5	6.5	2000	0.333
3	7	2	5000	0.279	18	7	6.5	6000	0.316
4	10	2	4000	0.345	19	10	6.5	5000	0.355
5	13	2	3000	0.368	20	13	6.5	4000	0.383
6	3	3.5	4000	0.26	21	3	8	5000	0.286
7	5	3.5	3000	0.274	22	5	8	4000	0.328
8	7	3.5	2000	0.297	23	7	8	3000	0.327
9	10	3.5	6000	0.372	24	10	8	2000	0.405
10	13	3.5	5000	0.386	25	13	8	6000	0.435
11	3	5	6000	0.24	26	3	3	4500	0.252
12	5	5	5000	0.259	27	5	5	3500	0.259
13	7	5	4000	0.283	28	7	7	4000	0.303
14	10	5	3000	0.301	29	10	7	5000	0.330
15	13	5	2000	0.367	30	7	5	3000	0.312

**Table 2:** Analysis of variance.

Source of Variance	Square Sum	DF	Mean Square	*F*	*p*
Normal force (A)	0.0350	1	0.0350	146.41	<0.0001
Feed speed (B)	0.0035	1	0.0035	14.70	0.0016
Spindle speed (C)	0.0004	1	0.0004	1.62	0.2223
AB	0.0002	1	0.0002	0.7858	0.3894
AC	0.0068	1	0.0068	28.48	<0.0001
BC	0.0008	1	0.0008	3.31	0.0888
A^2^	0.0022	1	0.0022	9.32	0.0081
B^2^	0.0035	1	0.0035	14.70	0.0016
C^2^	0.0027	1	0.0027	11.12	0.0045
Residual	0.0036	15	0.0002		
Model	0.0564	9	0.0063	26.21	<0.0001

## Data Availability

The original data supporting this study is included in the article.
